# Re-Evaluation of the Survival Paradox Between Stage IIB/IIC and Stage IIIA Colon Cancer

**DOI:** 10.3389/fonc.2020.595107

**Published:** 2020-11-18

**Authors:** Hongbo Li, Guangshun Fu, Wei Wei, Yong Huang, Zhenguang Wang, Tao Liang, Shuyun Tian, Honggang Chen, Wei Zhang

**Affiliations:** Department of General Surgery, Jiangdu People’s Hospital Affiliated to Yangzhou University Medical School, Yangzhou, China

**Keywords:** survival paradox, stage IIB/IIC, stage IIIA, colon cancer, T1N2a

## Abstract

**Objective:**

We conducted this large population-based study to re-evaluate the survival paradox between stage IIB/C and stage IIIA colon cancer based on the newest staging criteria.

**Methods:**

Colon cancer patients were recruited from the Surveillance, Epidemiology, and End Results (SEER) database using SEER*Stat software (version 8.3.4) with strict inclusion criteria. We used Chi-square test to compare categorical variables between patients diagnosed with stage IIB/IIC and stage IIIA colon cancer. Survival probabilities were then assessed using the Kaplan–Meier method. Cox proportional hazards models were used to analyze hazard ratios (HRs) and 95% confidence intervals (CIs) of clinicopathologic characteristics in stage IIB/IIC and stage IIIA colon cancer patients.

**Results:**

In the current study, a total of 9,227 eligible colon cancer patients were collected from the SEER database between 2010 and 2015. It was found that stage IIIA had 66.4% decreased risk of colon cancer-specific mortality compared with stage IIB (HR = 0.336, 95%CI = 0.286–0.394 for stage IIIA, P < 0.001, using stage IIB as the reference) after the adjustment for other known prognostic factors. And T1N2a colon cancer had significantly lower 5-year overall survival (OS) rate compared with T2N1 disease (74.7% vs. 57.1%, P = 0.018).

**Conclusions:**

Our study confirmed the existence of survival paradox between stage IIB/IIC and stage IIIA colon cancer based on the newest staging criteria. What is more, the subgroup analyses revealed that T1N2a had the least influence on the survival paradox. N2a colon cancer seemed to be associated with worse prognosis than T2 disease, which would give us a better understanding of tumor biology of colon cancer and be conducive to the refinement of individualized treatment regimens in stage III disease.

## Introduction

Colon cancer was one of the most common malignant tumors worldwide (1). And the American Joint Committee on Cancer (AJCC) TNM staging system was the most commonly used reference index for the guidance of treatment and the judgment of prognosis in many solid cancers. The AJCC staging system could accurately predict the prognosis of cancer patients, with lower stage cancers having better prognosis than higher stage cancers in most solid cancers (2). For colon cancer, however, a survival paradox could be observed between stage IIB/C (T4N0) and stage IIIA (T1-2N1, T1N2a) tumors in previous studies (3–7).

From the 6th to 7th editions of the AJCC staging system, T4 had been subdivided into T4a and T4b, and N1 had been subdivided into N1a, N1b, and N1c. However, no large population-based studies had been reported to evaluate the prognosis of subgroups in stage IIB/C and stage IIIA colon cancer behind the survival paradox according to the newest AJCC TNM staging criteria.

Several reasons had been reported to contribute to the inferior survival in stage IIB/IIC compared with that of stage IIIA, such as the lower use of systemic chemotherapy in stage IIB/IIC colon cancer patients and the stage migration due to inadequate retrieval of lymph nodes. We then conducted this large population-based study to evaluate the prognosis of different subgroups based on the newest staging criteria, together with inclusion of the retrieval of lymph nodes and the receipt of adjuvant chemotherapy, which we believed would contribute to a better understanding of the survival paradox between stage IIB/C and stage IIIA colon cancer.

## Materials and Methods

### Data Source

The Surveillance, Epidemiology, and End Results (SEER) program covered approximately 28% of the US population and was considered representative of the US in terms of cancer-related data. It collected de-identified data including cancer incidence, clinicopathological characteristics, treatment modalities, and survival from 18 participating population-based cancer registries annually (8). We then used SEER*Stat software (version 8.3.4, Surveillance Research Program, National Cancer Institute) to identify cases meeting the requirements of our study.

### Study Population

**Figure 1:**
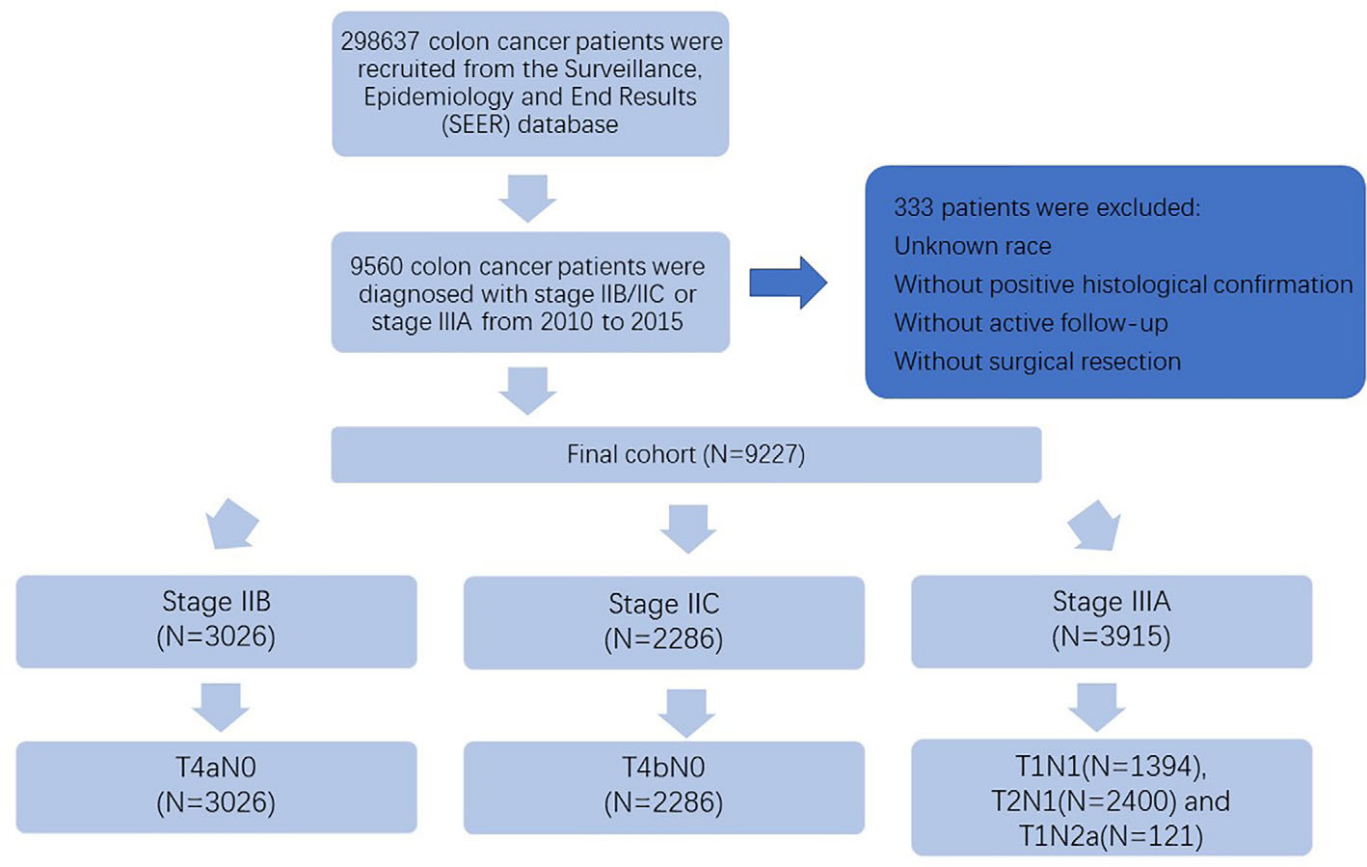
Flow diagram showed how eligible cases were selected from the SEER database.

Shown as **Figure 1**, at first, 298,637 colon cancer patients were recruited from the SEER database between 2004 and 2015. The present study aimed to conduct a detailed evaluation of survival paradox between stage IIB/IIC and stage IIIA colon cancer according to newest staging classification. Therefore, patients diagnosed before 2010 were excluded from the present study, only patients with complete information regarding the American Joint Committee on Cancer (AJCC) 7th TNM staging system and diagnosed with stage IIB/IIC or stage IIIA were retained. In addition, patients with unknown race, without positive histological confirmation, without active follow-up, or without surgical resection were excluded from our analyses. The final cohort included patients diagnosed with stage IIB, stage IIC, and stage IIIA, and we collected the relevant patient information including age (≤65 and >65 years), race (including white, black, and other), gender (including male and female), grade (including grade I/II, grade III/IV, and unknown), histology (including adenocarcinoma, and mucinous adenocarcinoma/signet ring cell carcinoma), No. of examined lymph nodes (<12 and ≥12), chemotherapy (no/unknown and yes), and TNM stage (stage IIB, stage IIC, and stage IIIA). Because we wanted to re-evaluated the survival paradox between stage IIB/IIC and stage IIIA colon cancer in detail, furtherly, all the cases were divided into five subgroups, including T4aN0, T4bN0, T1N1, T2N1, and T1N2a.

### Statistical Analysis

In our analyses, the outcomes variables of interest were colon cancer-specific survival (CCSS, from the time of diagnosis to the time of colon cancer-related death) and overall survival (OS, from the time of diagnosis to the time of death from any cause). First of all, in the present study, we used Chi-square test to compare categorical variables between patients diagnosed with stage IIB/IIC and stage IIIA colon cancer. Survival probabilities were then assessed using the Kaplan–Meier method, and the log-rank tests were used to evaluate any significant differences in CCSS and OS. Univariate and multivariate cox proportional hazards models were used to analyze hazard ratios (HRs) and 95% confidence intervals (CIs) of clinicopathologic characteristics for stage IIB/IIC and stage IIIA colon cancer patients. Only factors with a statistical significance (log rank, P < 0.20) in the univariate Cox analysis would be included in the multivariate Cox analyses. In our univariate analyses, clinicopathologic characteristics including age, race, gender, grade, histology, No. of examined lymph nodes, chemotherapy, and TNM stage were included in the multivariate Cox analyses. A two-sided p value less than 0.05 was considered statistically significant. Analyses were performed using SPSS version 23 statistical software (IBM Corporation).

## Results

### Patient Baseline Characteristics

In the current study, a total of 9,227 eligible colon cancer patients were collected from the SEER database between 2010 and 2015. The median follow-up duration was 33 months. Patient characteristics were listed in **Table 1**. Of the 9,227 patients diagnosed with stage IIB/IIC and stage IIIA colon cancer, 4,459 (48.3%) patients were female and 4,768 (51.7%) patients were male. A total of 3,897 (42.2%) patients were ≤65 years, and 5,330 (57.8%) patients were >65 years. Among these patients, 7,778 (84.3%) patients had enough lymph node retrieved, while 1,449 (15.7%) patients did not. The 3-year and 5-year CCSS rates of all the patients were 86.8% and 81.6%, respectively; The 3-year and 5-year OS rates in the SEER cohort were 73.7% and 63.2%, respectively. Based on the chi-squared test between stage IIB/IIC and stage IIIA colon cancer, stage IIIA was found to be associated with younger age (P < 0.001), black race (P < 0.001), male (P = 0.016), grade I/II (P < 0.001), adenocarcinoma (P < 0.001), low number of lymph nodes retrieved (P < 0.001), and the receipt of chemotherapy (P < 0.001), indicating that stage IIIA patients were more likely to be associated with some favorable clinicopathological characteristics (**Table 1**).

**Table 1:** Clinical features of stage IIB/IIC and stage IIIA colon cancer.

Characteristics	Number of patients (%)		*P*
	Stage IIB/IIC(N = 5,312)	Stage IIIA(N = 3,915)	
**Age (years)**			<0.001
≤**65**	2,002 (37.7)	1,895 (48.4)	
>**65**	3,310 (62.3)	2,020 (51.6)	
**Race**			<0.001
**White**	4,350 (81.9)	2,941 (75.1)	
**Black**	552 (10.4)	566 (14.5)	
**Other**	410 (7.7)	408 (10.4)	
**Gender**			0.016
**Male**	2,510 (47.3)	1,949 (49.8)	
**Female**	2,802 (52.7)	1,966 (50.2)	
**Grade**			<0.001
**Grade I/II**	3,928 (73.9)	3,139 (80.2)	
**Grade III/IV**	1,259 (23.7)	631 (16.1)	
**Unknown**	125 (2.4)	145 (3.7)	
**Histology**			<0.001
**Adenocarcinoma**	4,554 (85.7)	3,700 (94.5)	
**Mucinous adenocarcinoma/signet ring cell carcinoma**	758 (14.3)	215 (5.5)	
**No. of examined lymph nodes**			<0.001
**<12**	774 (14.6)	675 (17.2)	
**≥12**	4,538 (85.4)	3,240 (82.8)	
**Chemotherapy**			<0.001
**No/unknown**	3,450 (64.9)	1,457 (37.2)	
**Yes**	1,862 (35.1)	2,458 (62.8)	

### Survival Paradox Between Stage IIB/IIC and Stage IIIA Colon Cancer

**Figure 2:**
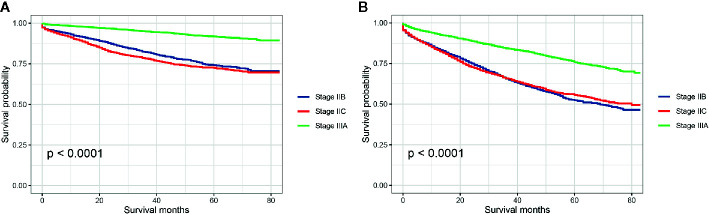
**(A)** Colon cancer-specific survival and **(B)** overall survival for stage IIB, stage IIC, and stage IIA colon cancer patients.

Stratified by AJCC TNM stage (stage IIB, stage IIC, and stage IIIA), Kaplan–Meier CCSS curves were shown in **Figure 2A**, and survival differences were estimated with log-rank tests. The CCSS rate of stage IIIA colon cancer patients was significantly higher than stage IIB, stage IIC colon cancer patients (3-year CCSS rates for stage IIB *vs.* stage IIC *vs.* stage IIIA, 82.2% *vs.* 78.2% *vs.* 94.9%, P < 0.0001; 5-year CCSS rates for stage IIB *vs.* stage IIC *vs.* stage IIIA, 74.2% *vs.* 72.5% *vs.* 91.9%, P < 0.0001; **Figure 2A**). Consistent with CCSS, the result of Kaplan–Meier OS analysis also showed that the OS rate of stage IIIA colon cancer patients was significantly higher than stage IIB, stage IIC colon cancer patients (3-year OS rates for stage IIB *vs.* stage IIC *vs.* stage IIIA, 65.6% *vs.* 65.8% *vs.* 84.4%, P < 0.0001; 5-year OS rates for stage IIB vs. stage IIC *vs.* stage IIIA, 52.4% *vs.* 55.6% *vs.* 75.8%, P < 0.0001; **Figure 2B**).

We also carried out univariate and multivariate Cox proportional hazards analyses to evaluate potential risk factors associated with the CCSS and the CCSS difference between stage IIB/IIC and stage IIIA colon cancer. All the clinicopathologic characteristics with prognostic significance were included in multivariate Cox proportional hazards analyses and the result of multivariate analyses was shown in **Table 2**: age [hazard ratio (HR) = 1.524, 95% confidence interval (CI) = 1.338–1.736 for >65 years, P < 0.001, using ≤65 years as the reference], race (HR = 1.429, 95%CI = 1.214–1.681 for black race; HR = 0.956, 95%CI = 0,773–1.183 for other, P < 0.001, using white race as the reference), grade (HR = 1.349, 95%CI = 1.187–1.533 for grade III/IV; HR = 1.250, 95%CI = 0.889–1.758 for unknown, P < 0.001, using grade I/II as the reference), No. of examined lymph nodes (HR = 0.530, 95%CI = 0.464–0.606 for ≥12 resected lymph nodes, P < 0.001, using <12 lymph nodes as the reference), and chemotherapy (HR = 0.595, 95%CI = 0.522–0.678 for the receipt of chemotherapy, P < 0.001, using no chemotherapy as the reference) were independently associated with the risk of colon cancer-specific mortality. What is more, it was found that stage IIIA had 66.4% decreased risk of colon cancer-specific mortality compared with stage IIB, and stage IIC had 32.4% increased risk of colon cancer-specific mortality compared with stage IIB (HR = 1.324, 95%CI = 1.169–1.500 for stage IIC; HR = 0.336, 95%CI = 0.286–0.394 for stage IIIA, P < 0.001, using stage IIB as the reference) after the adjustment for other relevant covariables.

**Table 2:** Cox regression analyses of factors associated with CSS.

Variable	Univariate analyses		Multivariate analyses	
	HR (95%CI)	*P*	HR (95%CI)	*P*
**Stage**		<0.001		<0.001
**Stage IIB**	1		1	
**Stage IIC**	1.199 (1.059–1.356)	0.004	1.324 (1.169–1.500)	<0.001
**Stage IIIA**	0.284 (0.243–0.331)	<0.001	0.336 (0.286–0.394)	<0.001
**Age (years)**		<0.001		<0.001
**≤65**	1		1	
**>65**	1.954 (1.730–2.206)		1.524 (1.338–1.736)	
**Race**		0.035		<0.001
**White**	1		1	
**Black**	1.161 (0.988–1.364)	0.069	1.429 (1.214–1.681)	<0.001
**Other**	0.840 (0.680–1.038)	0.107	0.956 (0,773–1.183)	0.680
**Gender**		0.001		0.136
**Male**	1		1	
**Female**	1.210 (1.081–1.353)		1.090 (0.973–1.221)	
**Grade**		<0.001		<0.001
**Grade I/II**	1		1	
**Grade III/IV**	1.528 (1.346–1.734)	<0.001	1.349 (1.187–1.533)	<0.001
**Unknown**	1.079 (0.770–1.513)	0.659	1.250 (0.889–1.758)	0.199
**Histology**		0.017		0.488
**Adenocarcinoma**	1		1	
**Mucinous adenocarcinoma/signet ring cell carcinoma**	1.231 (1.038–1.459)		0.941 (0.792–1.118)	
**No. of examined lymph nodes**		<0.001		<0.001
**<12**	1		1	
**≥12**	0.598 (0.523–0.682)		0.530 (0.464–0.606)	
**Chemotherapy**		<0.001		<0.001
**No/unknown**	1		1	
**Yes**	0.407 (0.360–0.459)		0.595 (0.522–0.678)	

### Further Analyses of Survival Paradox Between Stage IIB/IIC and Stage IIIA Colon Cancer

**Figure 3:**
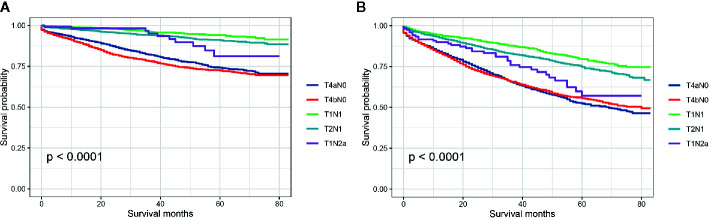
**(A)** Colon cancer-specific survival and **(B)** overall survival for stage T4aN0, stage T4bN0, stage T1N1, stage T2N1, and stage T1Na colon cancer patients.

Then, we further investigated which subgroup would contribute to survival paradox between stage IIB/IIC and stage IIIA colon cancer. Stratified by detailed stage (T4aN0, T4bN0, T1N1, T2N1, and T1Na), Kaplan–Meier CCSS curves were shown in **Figure 3A**, and survival differences between different subgroups were estimated with log-rank tests: 5-year CCSS rates for T4aN0 *vs.* T4bN0 *vs.* T1N1 *vs.* T2N1 *vs.* T1N2a, 74.2% *vs.* 72.5% *vs.* 94.2% *vs.* 91.0% *vs.* 81.2% (P < 0.0001), indicating that T1N2a colon cancer had inferior CCSS compared with T2N1 colon cancer though the survival difference did not achieve statistical significance (P = 0.406); Kaplan–Meier OS curves were shown in **Figure 3B**, and survival differences between different subgroups were estimated with log-rank tests: 5-year OS rates for T4aN0 *vs.* T4bN0 *vs.* T1N1 *vs.* T2N1 *vs.* T1N2a, 52.4% *vs.* 55.6% *vs.* 79.4% *vs.* 74.7% *vs.* 57.1% (P < 0.0001), and T1N2a colon cancer had significantly inferior OS compared with T2N1 colon cancer (P = 0.018).

Univariate and multivariate Cox proportional hazards analyses were carried out to evaluate potential risk factors associated with the CCSS and the CCSS differences between different subgroups (T4aN0, T4bN0, T1N1, T2N1, and T1Na). It was found that T2N1 had a 25.7% decreased risk of colon cancer-specific mortality compared with T1N2a (HR = 0.743, 95%CI = 0.392–1.408 for T2N1, using T1N2a as the reference) after the adjustment for other relevant covariables, though the survival difference did not achieve statistical significance (P = 0.362 **Table 3**).

**Table 3:** Cox regression analyses of factors associated with CSS (including T4aN0, T4bN0, T1N1, T2N1, and T1Na).

Variable	Univariate analyses		Multivariate analyses	
	HR (95%CI)	*P*	HR (95%CI)	*P*
**Stage**		<0.001		<0.001
**T4aN0**	1		1.956 (1.044–3.665)	0.036
**T4bN0**	1.199 (1.059–1.356)	0.004	2.588 (1.382–4.848)	0.003
**T1N1**	0.204 (0.156–0.267)	<0.001	0.478 (0.244–0.934)	0.031
**T2N1**	0.325 (0.272–0.388)	<0.001	0.743 (0.392–1.408)	0.362
**T1N2a**	0.421 (0.225–0.786)	0.007	1	
**Age (years)**		<0.001		<0.001
**≤65**	1		1	
**>65**	1.954 (1.730–2.206)		1.511 (1.326–1.721)	
**Race**		0.035		<0.001
**White**	1		1	
**Black**	1.161 (0.988–1.364)	0.069	1.422 (1.208–1.673)	<0.001
**Other**	0.840 (0.680–1.038)	0.107	0.953 (0.770–1.180)	0.660
**Gender**		0.001		0.129
**Male**	1		1	
**Female**	1.210 (1.081–1.353)		1.092 (0.975–1.223)	
**Grade**		<0.001		<0.001
**Grade I/II**	1		1	
**Grade III/IV**	1.528 (1.346–1.734)	<0.001	1.342 (1.181–1.524)	<0.001
**Unknown**	1.079 (0.770–1.513)	0.659	1.298 (0.922–1.826)	0.135
**Histology**		0.017		0.464
**Adenocarcinoma**	1		1	
**Mucinous adenocarcinoma/signet ring cell carcinoma**	1.231 (1.038–1.459)		0.938 (0.790–1.114)	
**No. of examined lymph nodes**		<0.001		<0.001
**<12**	1		1	
**≥12**	0.598 (0.523–0.682)		0.526 (0.460–0.601)	
**Chemotherapy**		<0.001		<0.001
**No/unknown**	1		1	
**Yes**	0.407 (0.360–0.459)		0.595 (0.522–0.678)	

## Discussion

The AJCC staging system contained information about the tumor status at diagnosis which could assist clinicians to predict survival, impart prognostic information, and give the guidance to select the most effective treatments. As early as 2000, the colorectal working group proposed to subdivide T4 into T4a (tumor penetrated the surface of the visceral peritoneum) and T4b (tumor directly invaded or was histologically adherent to other organs or structures) according to the absence or presence of tumor involving the surface of the specimen based on the evidence that previous study had found that peritoneal involvement had an adverse outcome (9, 10). Then, the 7^th^ edition AJCC TNM staging system published in 2010 subdivided T4 into T4a and T4b and further divided N1 into N1a (metastasis in 1 node), N1b (metastasis in 2–3 nodes) and N1c (the presence of tumor deposit, there was no regional lymph node metastasis), and N2 into N2a (metastasis in 4–6 nodes) and N2b (metastasis in ≥7 nodes). Therefore, stage II colon cancer was subdivided into IIA (T3N0), IIB (T4aN0), or IIC (T4bN0) and stage III colon cancer became IIIA (T1-2 N1, T1N2a), IIIB (T3-4 N1, T2-3N2a, T1-2N2b), and IIIC (T4aN2a, T3-T4aN2b, T4bN1-2) (2, 11, 12). And the eighth AJCC TNM staging system was the same as seventh staging system in regards to stage II and stage III colon cancer. Although the survival paradox between stage IIB/IIC and stage IIIA colon cancer had long been known, few population-based studies reported this phenomenon based on the newest AJCC TNM staging system or evaluated prognosis of subgroups in stage IIB/IIC and stage IIIA to further reveal the survival paradox in colon cancer (2–7).

In our analyses, it was found that the CCSS rate of stage IIIA colon cancer patients was significantly higher than stage IIB, stage IIC colon cancer patients (5-year CCSS rates for stage IIB *vs.* stage IIC *vs.* stage IIIA, 74.2% *vs.* 72.5% *vs.* 91.9%). Similarly, 5-year OS rates of stage IIB, stage IIC and stage IIIA were 52.4%, 55.6%, and 75.8%, respectively, which was consistent with previous report by Edge et at. (2) that the 5-year OS rate for patients with stage IIIA was approximately 70% *vs.* 46–61% for stage IIB/C. More importantly, we also conduct multivariate analyses to exclude the possibility of the influence of other prognostic factors including age, race, gender, grade, histology, No. of examined lymph nodes, and the receipt of chemotherapy. It was found that stage IIIA had 66.4% decreased risk of colon cancer-specific mortality compared with stage IIB. In other words, stage IIB/C (T4N0) colon cancer had worse prognosis compared with stage IIIA (T1-2 N1, T1N2a) even after adjusting for the number of lymph nodes retrieved and the receipt of adjuvant chemotherapy, which was in agreement with previous study and once again demonstrated the existence of survival paradox between stage IIB/IIC and stage IIIA colon cancer (3).

Previous studies had suggested that the poor survivals of T4N0 might attribute to the following factors: preferential administration of chemotherapy for stage IIIA compared with T4N0 disease (while the present study had shown that 35.1% of T4N0 colon cancer patients would receive adjuvant chemotherapy compared with 62.8% of stage IIIA colon cancer patients); T4N1 colon cancer was understaged as T4N0 due to inadequate retrieval of lymph nodes (while 85.4% of T4N0 colon cancer patients had enough retrieval of lymph nodes compared with 82.8% of stage IIIA colon cancer patients in our analyses) and biologically more aggressive tumors in T4N0 (13, 14). In 2016, Quyen and his colleagues (3) carried out a retrospective analysis and found that the survival paradox between stage IIB/IIC and stage IIIA colon cancer cannot be entirely explained by inadequate lymph nodes retrieved and lack of receipt of adjuvant chemotherapy, which was consistent with the current study. A previous study showed that T4N0 colon cancers were associated with higher proportion of MSI-H and poor histological grade, indicating that T4N0 carcinomas might have different entity of tumor biology from T1-2N1 disease (4, 15).

In 2016, Quyen et al. (6) reported that stage IIB/C were associated with a greater proportion of positive margins (19%) than did stage IIIA (1%; P <.0001), from this they believed that positive surgical margins might contribute to the survival paradox between stage IIB/C and stage IIIA colon cancer patients. Our study also showed that stage IIB/IIC colon cancer was more likely to be associated with grade III/IV compared with stage IIIA disease (23.7% *vs.* 16.1%, P < 0.001), which could add new evidence supporting the above hypothesis.

Although the fact that the use of adjuvant chemotherapy had been widely accepted as the routine treatment for patients with stage III colon cancer and stage IIB/IIC colon cancer had significant poor survivals compared with stage IIIA disease, some researchers have suggested that the efficacy of adjuvant chemotherapy in T4 disease was not significant (16–21). Therefore, future studies were still needed to investigate the necessity of intensive chemotherapy in T4 colon cancer. In further exploration of the present study, Kaplan–Meier survival curves showed the 5-year CCSS rates (T4aN0 *vs.* T4bN0 *vs.* T1N1 *vs.* T2N1 *vs.* T1N2a, 74.2% *vs.* 72.5% *vs.* 94.2% *vs.* 91.0% *vs.* 81.2%) and 5-year OS rates (5-year OS for T4aN0 *vs.* T4bN0 *vs.* T1N1 *vs.* T2N1 *vs.* T1Na, 52.4% *vs.* 55.6% *vs.* 79.4% *vs.* 74.7% *vs.* 57.1%) of different subgroups in stage IIB/IIC and stage IIIA colon cancer, indicating that T1N2a colon cancer had inferior CCSS (P = 0.406) and OS (P = 0.018) compared with T2N1 colon cancer though the CCSS difference between T1N2a and T2N1 colon cancer did not achieve statistical significance.

The results of multivariate analyses in the present study also showed the similar result that T2N1 colon cancer had a 25.7% decreased risk of colon cancer-specific mortality compared with T1N2a (HR = 0.743, 95%CI = 0.392–1.408 for T2N1, using T1N2a as the reference) after the adjustment for other relevant covariables, though the survival difference did not achieve statistical significance (P = 0.362). The above findings also indicated the inconsistency of subgroups in stage IIIA colon cancer, especially between stage T2N1 and stage T1N2a though they were both classified as stage IIIA. And N2a seemed to be a stronger factor for poor prognosis than T2 stage, the increase of one positive lymph node seemed to be a worse indicator of survival compared with the penetration of tumor from submucosa to muscular layer. That the CCSS difference between T1N2a and T2N1 colon cancer did not achieve statistical significance might be because of the small sample size of T1N2a (N = 121).

The main strengths of the present study were that, as far as we know, this was the first population-based analysis to evaluate prognosis of detailed subgroups in stage IIB/IIC and stage IIIA to further reveal the survival paradox in colon cancer based on the newest staging criteria and a large population. The finding that N2a colon cancer seemed to be a worse prognostic factor than T2 disease revealed the inconsistence in stage IIIA colon cancer and T1N2a had the least influence on the survival paradox between stage IIB/IIC and stage IIIA, which could give us a better understanding of tumor biology of colon cancer and be conducive to the refinement of individualized treatment regimens in stage III disease.

However, this study had two limitations. On the one hand, information on the surgical margin status, molecular and genetic markers that were confirmed as prognostic factors of colon cancer were lacking because of the limitation of the SEER database (22–24). On the other hand, our research was a retrospective type of study with inherent deficiencies that could lead to confusion or observer bias, and future research could overcome this problem by the use of a prospective diary. In addition, external validation is missing because of insufficient eligible patients in our center.

In conclusion, our study confirmed the presence of survival paradox between stage IIB/IIC and stage IIIA colon cancer based on the newest staging criteria. What is more, the subgroup analyses revealed the inconsistence in stage IIIA colon cancer and T1N2a had the least influence on the survival paradox. N2a colon cancer seemed to be a worse prognostic factor than T2 disease, which would give us a better understanding of tumor biology of colon cancer and be conducive to the refinement of individualized treatment regimens in stage III disease.

## Data Availability Statement

Publicly available datasets were analyzed in this study. This data can be found here: Surveillance, Epidemiology, and End Results (SEER) database (https://seer.cancer.gov/).

## Author Contributions

HL, GF, WW, and WZ conceived and designed the study. YH, ZW, and TL collected and analyzed the data. HL, TL, ST, and HC performed the statistical analysis. HL and GF wrote the first draft of the manuscript. HC and WZ revised the final data and the manuscript. All the authors contributed to the article and approved the submitted version.

## Conflict of Interest

The authors declare that the research was conducted in the absence of any commercial or financial relationships that could be construed as a potential conflict of interest.

## References

[1] Torre LA Bray F Siegel RL Ferlay J Lortet-Tieulent J Jemal A Global cancer statistics, 2012. CA: Cancer J Clin (2015) 65(2):87–108. 10.3322/caac.21262 25651787

[2] Edge SB Compton CC The American Joint Committee on Cancer: the 7th edition of the AJCC cancer staging manual and the future of TNM. Ann Surg Oncol (2010) 17(6):1471–4. 10.1245/s10434-010-0985-4 20180029

[3] Chu QD Zhou M Medeiros KL Peddi P Kavanaugh M Wu X-C Poor survival in stage IIB/C (T4N0) compared to stage IIIA (T1-2?N1, T1N2a) colon cancer persists even after adjusting for adequate lymph nodes retrieved and receipt of adjuvant chemotherapy. BMC Cancer (2016) 16(1):460. 10.1186/s12885-016-2446-3 27412163 PMC4944507

[4] Kim MJ Jeong S-Y Choi S-j Ryoo S-B Park JW Park KJ Survival Paradox Between Stage IIB/C (T4N0) and Stage IIIA (T1-2N1) Colon Cancer. Ann Surg Oncol (2015) 22(2):505–12. 10.1245/s10434-014-3982-1 25145501

[5] Lorenzon L Balducci G Ferri M Sub-Staging Colorectal Cancers and Adjuvant Treatments. J Am Coll Surg (2015) 220(3):379–81. 10.1016/j.jamcollsurg.2014.12.005 25700909

[6] Chu QD Zhou M Medeiros K Peddi P Positive surgical margins contribute to the survival paradox between patients with stage IIB/C (T4N0) and stage IIIA (T1-2N1, T1N2a) colon cancer. Surgery (2016) 160(5):1333–43. 10.1016/j.surg.2016.05.028 27425043

[7] Kim HS Kim KM Lee SB Kim GR Han YD Cho MS Clinicopathological and biomolecular characteristics of stage IIB/IIC and stage IIIA colon cancer: Insight into the survival paradox. J Surg Oncol (2019) 120(3):423–30. 10.1002/jso.25515 31134644

[8] Cai Y Cheng G Lu X Ju H Zhu X The re-evaluation of optimal lymph node yield in stage II right-sided colon cancer: is a minimum of 12 lymph nodes adequate? Int J Colorectal Dis (2020) 35(4):623–31. 10.1007/s00384-019-03483-z 31996986

[9] Compton C Fenoglio-Preiser CM Pettigrew N Fielding LP American Joint Committee on Cancer Prognostic Factors Consensus Conference: Colorectal Working Group. Cancer (2000) 88(7):1739–57. 10.1002/(SICI)1097-0142(20000401)88:7<1739::AID-CNCR30>3.0.CO;2-T 10738234

[10] Shepherd NA Baxter KJ Love SB The prognostic importance of peritoneal involvement in colonic cancer: a prospective evaluation. Gastroenterology (1997) 112(4):1096–102. 10.1016/S0016-5085(97)70119-7 9097991

[11] Hari DM Leung AM Lee JH Sim MS Vuong B Chiu CG AJCC Cancer Staging Manual 7th edition criteria for colon cancer: do the complex modifications improve prognostic assessment? J Am Coll Surg (2013) 217(2):181–90. 10.1016/j.jamcollsurg.2013.04.018 PMC4657944 23768788

[12] Tong GJ Zhang GY Liu J Zheng ZZ Chen Y Niu PP Comparison of the eighth version of the American Joint Committee on Cancer manual to the seventh version for colorectal cancer: A retrospective review of our data. World J Clin Oncol (2018) 9(7):148–61. 10.5306/wjco.v9.i7.148 PMC6230917 30425940

[13] O’Connell JB Maggard MA Ko CY Colon cancer survival rates with the new American Joint Committee on Cancer sixth edition staging. J Natl Cancer Inst (2004) 96(19):1420–5. 10.1093/jnci/djh275 15467030

[14] Jeong SY Chessin DB Schrag D Riedel E Wong WD Guillem JG Re: Colon cancer survival rates with the new American Joint Committee on Cancer sixth edition staging. J Natl Cancer Inst (2005) 97(22):1705–6. 10.1093/jnci/dji383 16288127

[15] Benedix F Meyer F Kube R Kropf S Kuester D Lippert H Influence of anatomical subsite on the incidence of microsatellite instability, and KRAS and BRAF mutation rates in patients with colon carcinoma. Pathol Res Pract (2012) 208(10):592–7. 10.1016/j.prp.2012.07.003 22898351

[16] O'Connor ES Greenblatt DY LoConte NK Gangnon RE Liou JI Heise CP Adjuvant chemotherapy for stage II colon cancer with poor prognostic features. J Clin Oncol (2011) 29(25):3381–8 10.1200/JCO.2010.34.3426 PMC3164243 21788561

[17] Min KK Won DD Sun MP Kim T Lee IK Effect of Adjuvant Chemotherapy on Stage II Colon Cancer: Analysis of Korean National Data. Cancer Res Treat (2017) 50(4):1149–63. 10.4143/crt.2017.194 PMC6192938 29216709

[18] Sargent D Sobrero A Grothey A O'Connell MJ Buyse M Andre T Evidence for cure by adjuvant therapy in colon cancer: observations based on individual patient data from 20,898 patients on 18 randomized trials. J Clin Oncol (2009) 27(6):872–7. 10.1200/JCO.2008.19.5362 PMC2738431 19124803

[19] Group QC Adjuvant chemotherapy versus observation in patients with colorectal cancer: a randomised study. Lancet (2007) 370(9604):0–2029. 10.1016/S0140-6736(07)61866-2 18083404

[20] André T Boni C Mounedji-Boudiaf L Navarro M Tabernero J Hickish T Multicenter International Study of Oxaliplatin/5-Fluorouracil/Leucovorin in the Adjuvant Treatment of Colon Cancer (MOSAIC) Investigators. Oxaliplatin, fluorouracil, and leucovorin as adjuvant treatment for colon cancer. N Engl J Med (2004) 350(23):2343–51. 10.1056/NEJMoa032709 10.1056/NEJMoa032709 15175436

[21] Liu Q Huang Y Luo D Zhang S Cai S Li Q Evaluating the Guiding Role of Elevated Pretreatment Serum Carcinoembryonic Antigen Levels for Adjuvant Chemotherapy in Stage IIA Colon Cancer: A Large Population-Based and Propensity Score-Matched Study. Front Oncol (2019) 9:37. 10.3389/fonc.2019.00037 30815388 PMC6381003

[22] Chouhan H Sammour T Thomas ML Moore JW The interaction between BRAF mutation and microsatellite instability (MSI) status in determining survival outcomes after adjuvant 5FU based chemotherapy in stage III colon cancer. J Surg Oncol (2018) 118(8):1311–7. 10.1002/jso.25275 30399198

[23] Frouws MA Reimers MS Swets M Bastiaannet E Prinse B van Eijk R The Influence of BRAF and KRAS Mutation Status on the Association between Aspirin Use and Survival after Colon Cancer Diagnosis. PloS One (2017) 12(1):e0170775. 10.1371/journal.pone.0170775 28125730 PMC5268402

[24] Callebout E Ribeiro SM Laurent S De Man M Ferdinande L Claes KBM Long term response on Regorafenib in non-V600E BRAF mutated colon cancer: a case report. BMC Cancer (2019) 19(1):567. 10.1186/s12885-019-5763-5 31185985 PMC6560823

